# SRC tyrosine kinase activates the YAP/TAZ axis and thereby drives tumor growth and metastasis

**DOI:** 10.1074/jbc.RA118.004364

**Published:** 2018-12-17

**Authors:** John M. Lamar, Yuxuan Xiao, Emily Norton, Zhi-Gang Jiang, Genevieve M. Gerhard, Simrin Kooner, Janine S. A. Warren, Richard O. Hynes

**Affiliations:** From the ‡ Department of Molecular and Cellular Physiology, Albany Medical College, Albany, New York 12208 and; the § Koch Institute for Integrative Cancer Research,; ‖ Department of Biology, and; ¶ Howard Hughes Medical Institute, Massachusetts Institute of Technology, Cambridge, Massachusetts 02139

**Keywords:** Yes-associated protein (YAP), cancer, metastasis, Src, LATS (Warts, Wts), Hippo pathway, Non-receptor tyrosine kinase (nRTK), GIT1, TAZ, transcriptional regulator

## Abstract

When properly employed, targeted therapies are effective cancer treatments. However, the development of such therapies requires the identification of targetable drivers of cancer development and metastasis. The expression and nuclear localization of the transcriptional coactivators Yes-associated protein (YAP) and transcriptional co-activator with PDZ-binding motif (TAZ) are increased in many human cancers, and experimental evidence indicates that aberrant YAP or TAZ activation drives tumor formation and metastasis. Although these findings make YAP and TAZ appealing therapeutic targets, both have important functions in adult tissues, so directly targeting them could cause adverse effects. The identification of pathways active in cancer cells and required for YAP/TAZ activity could provide a way to inhibit YAP and TAZ. Here, we show that SRC proto-oncogene, nonreceptor tyrosine kinase (SRC) is an important driver of YAP/TAZ activity in human breast cancer and melanoma cells. SRC activation increased YAP/TAZ activity and the expression of YAP/TAZ-regulated genes. In contrast, SRC inhibition or knockdown repressed both YAP/TAZ activity and the expression of YAP/TAZ-regulated genes. We also show that SRC increases the activity of YAP and TAZ by repressing large tumor suppressor homolog (LATS), and we identify the GTPase-activating protein GIT ArfGAP 1 (GIT1) as an SRC effector that regulates both YAP and TAZ. Importantly, we demonstrate that SRC-mediated YAP/TAZ activity promotes tumor growth and enhances metastasis and that SRC-dependent tumor progression depends, at least in part, on YAP and TAZ. Our findings suggest that therapies targeting SRC could help manage some YAP/TAZ-dependent cancers.

## Introduction

The vast majority of cancer-related deaths are caused by metastasis, and few effective treatments exist for patients with metastatic disease. To develop more effective targeted therapies, we need to identify proteins required for metastasis formation and growth and understand how the pathways that regulate these proteins become dysfunctional in cancer cells. During tumor growth and metastasis, cancer cells encounter a constantly changing microenvironment, and cancer cells must acquire the ability to interpret and respond to changing cues that they receive from that microenvironment. As such, proteins that regulate gene expression in response to numerous microenvironmental cues, like the transcriptional co-activators Yes-associated protein (YAP)^3^ and transcriptional co-activator with PDZ-binding motif (TAZ), are well positioned to influence cancer progression and metastasis. Consistently, YAP and TAZ have both emerged as drivers of cancer development, tumor progression, and metastasis, and preventing aberrant YAP/TAZ activity appears to be a promising therapeutic strategy. Therefore, identifying pathways that promote aberrant YAP/TAZ activity in cancer cells could facilitate the development of more effective targeted therapies.

YAP and TAZ are the downstream effectors of the mammalian Hippo pathway, which was initially elucidated in *Drosophila melanogaster* and is largely conserved in mammals and other vertebrates (1). As in flies, the mammalian Hippo pathway consists of a core kinase cascade in which activated mammalian sterile 20-like kinase 1 or 2 (MST1 or MST2) binds and phosphorylates the scaffold protein Salvador homolog 1 (SAV1) (2, 3). The active MST–SAV1 complex then phosphorylates and activates one or both of the downstream kinases large tumor suppressor homolog 1 and 2 (LATS1 and LATS2) as well the scaffold proteins MOB kinase activator 1A and 1B (MOB1A and MOB1B) (4, 5). The active LATS–MOB complex then phosphorylates and represses YAP and TAZ. LATS-mediated phosphorylation of YAP on serine 127 or TAZ on serine 89 promotes 14-3-3 binding and cytoplasmic sequestration (6–8), whereas phosphorylation of serine 381 of YAP or serine 311 of TAZ promotes subsequent phosphorylation by casein kinase I δ/ϵ and recruitment of the E3 ubiquitin ligase SCF(β-TRCP), leading to proteasomal degradation (9, 10). Nonphosphorylated YAP and TAZ can enter the nucleus and partner with other transcription factors (11) to promote gene expression. Although YAP and TAZ can partner with several transcription factors, the TEA domain family members (TEADs) 1–4 mediate many YAP/TAZ-dependent processes in both normal and cancerous cells (11–14). A long and rapidly growing list of proteins and pathways can regulate YAP and TAZ in response to altered microenvironmental cues (for reviews, see Refs. 15–21).

It is now clear that dysregulation of the Hippo-YAP/TAZ pathway is an important driver of cancer development, tumor progression, and metastasis. There is abundant experimental evidence from both cell-culture and mouse models showing that inappropriate YAP/TAZ activity promotes tumor formation and growth and enhances tumor progression (22–24). YAP/TAZ activation also drives metastasis. Indeed, since our initial finding that YAP activation is sufficient to drive cancer metastasis (25), there have been several studies in a variety of cancer types that found that YAP or TAZ activation promotes metastasis (reviewed in Refs. 22 and 23). Collectively, these studies show that YAP and TAZ activation enhances just about every step of the metastatic cascade. Furthermore, analysis of human cancer samples has overwhelmingly demonstrated that the expression and/or activity of YAP or TAZ is increased in a high percentage of human cancers compared with corresponding normal tissue (reviewed in Refs. 1, 23, and 24) and that this elevated activity is strongly associated with poor outcome and reduced survival (26, 27). Intriguingly, although genetic alterations in the core Hippo cascade and amplifications in YAP and TAZ do exist in human cancers, the frequency of these events is not high enough to explain the elevated YAP/TAZ activity commonly observed. This suggests that other pathways that are aberrantly activated in cancer cells promote YAP/TAZ activation to drive tumor growth and metastasis. Identification of these pathways could facilitate the development of targeted therapies for use in YAP/TAZ-driven cancers.

Here we demonstrate that SRC is an important driver of YAP/TAZ activity in several breast cancer and melanoma cell lines and show that SRC-mediated YAP/TAZ activation is important for tumor growth and metastasis. We found that SRC activates YAP and TAZ by repressing LATS but that SRC effector pathways known to regulate YAP and TAZ in other cell types are not playing a significant role in these cancer cells. Instead, we show that GTPase-activating protein GIT ArfGAP 1 (GIT1) is an SRC effector that regulates YAP/TAZ activity in both melanoma and breast cancer cells. Our findings, in combination with other recent publications, show that SRC can activate YAP and TAZ through multiple distinct mechanisms and suggest that SRC is an important upstream signaling node that controls YAP and TAZ. This reveals SRC as a potential therapeutic target for the treatment of YAP/TAZ-driven cancers.

## Results

### SRC activation promotes YAP/TAZ transcriptional activity and target gene expression

**Figure 1.**
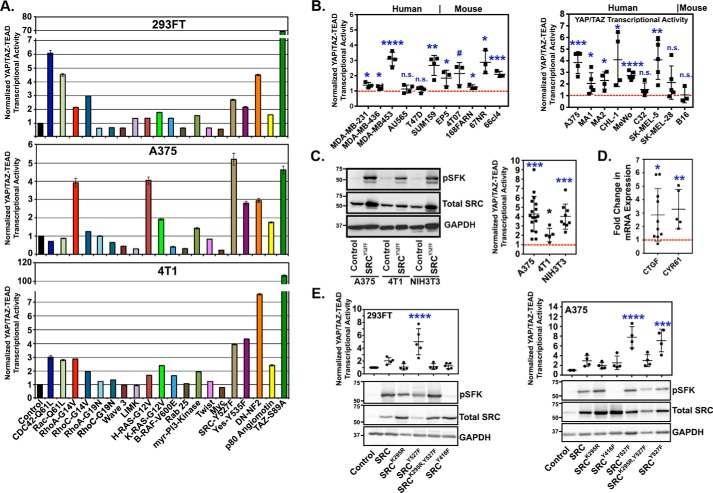
**SRC activation promotes YAP/TAZ activity and target gene expression in multiple breast cancer and melanoma cell lines.** *A*, cell lines were transiently co-transfected with the indicated constructs and YAP/TAZ-TEAD luciferase reporter vectors, and 48 h later, YAP/TAZ-TEAD transcriptional activity was assayed (*n* = 4 replicates from one experiment). *B*, YAP/TAZ-TEAD transcriptional activity was assayed in the indicated human and mouse breast cancer and melanoma cells following transient transfection with either control vector or activated SRC^Y527F^. *C*, cells were stably transduced with either control vector or SRC^Y527F^ and then assayed by Western blotting (*left*) or for YAP/TAZ-TEAD transcriptional activity (*right*). *D*, cells from *C* were assayed by qPCR for CTGF and CYR61 mRNA. *E*, cells transiently (*left*) or stably (*right*) expressing control vector or the indicated SRC mutants were assayed by Western blotting or for YAP/TAZ-TEAD transcriptional activity. Scatter plots show mean ± S.D. (*error bars*), where each *dot* is an independent experiment in which the SRC^Y527F^ cells were converted to -fold increase over control cells, which are indicated by the *red dotted line*. Statistical significance was tested using Student's *t* test (*B–D*) and one-way ANOVA with Tukey's post hoc test (*E*). *n.s.*, *p* > 0.055; #, *p* ≤ 0.055; *, *p* ≤ 0.05; **, *p* ≤ 0.01; ***, *p* ≤ 0.001; ****, *p* ≤ 0.0001.

As discussed above, it is clear that increased YAP/TAZ activity is prevalent in human cancer, but it remains unclear exactly how YAP and TAZ become activated in most cancer types. To identify oncogenic pathways that activate YAP and TAZ, we performed a screen in which several cancer-associated genes were individually co-transfected into cells with a YAP/TAZ-TEAD transcriptional reporter construct. Dominant-negative forms of NF2 and angiomotin, which negatively regulate YAP and TAZ, were also included as controls because these constructs are known to promote YAP/TAZ activity (10, 11). Multiple cancer-associated proteins, including several that have since been shown by others to regulate YAP or TAZ (RhoA, Cdc42, Ras, and phosphoinositide 3-kinase (PI3K)) (28–32), increased YAP/TAZ transcriptional activity (Fig. 1*A*). This demonstrates that our approach can effectively identify biologically relevant YAP/TAZ regulators. We focused on SRC, given its known roles in cancer progression and metastasis (33–42) and because it is frequently up-regulated or activated in human cancers (39, 42–46). Expression of a constitutively active SRC mutant (SRC^Y527F^) promoted YAP/TAZ transcriptional activity in the majority of the cell lines we tested, including several human and mouse breast cancer and melanoma cell lines (Fig. 1*B*). Stable expression of SRC^Y527F^ also promoted YAP/TAZ transcriptional activity (Fig. 1*C*) and increased the mRNA expression of the known YAP/TAZ-regulated genes connective tissue growth factor (CTGF) and cysteine-rich angiogenic inducer 61 (CYR61) (Fig. 1*D*). Overexpression of WT SRC also increases YAP/TAZ transcriptional activity, although to a lesser extent than SRC^Y527F^ (Fig. 1*E*). Conversely, kinase-dead SRC (SRC^K295R^) and dominant-negative SRC (SRC^Y416F^) failed to enhance YAP/TAZ transcriptional activity (Fig. 1*E*), suggesting that SRC kinase activity is required. These results show that SRC activation promotes YAP/TAZ transcriptional activity and the expression of YAP/TAZ-regulated genes in a kinase-dependent manner.

### SRC activation is required for YAP/TAZ transcriptional activity and the induction of YAP/TAZ target genes

**Figure 2.**
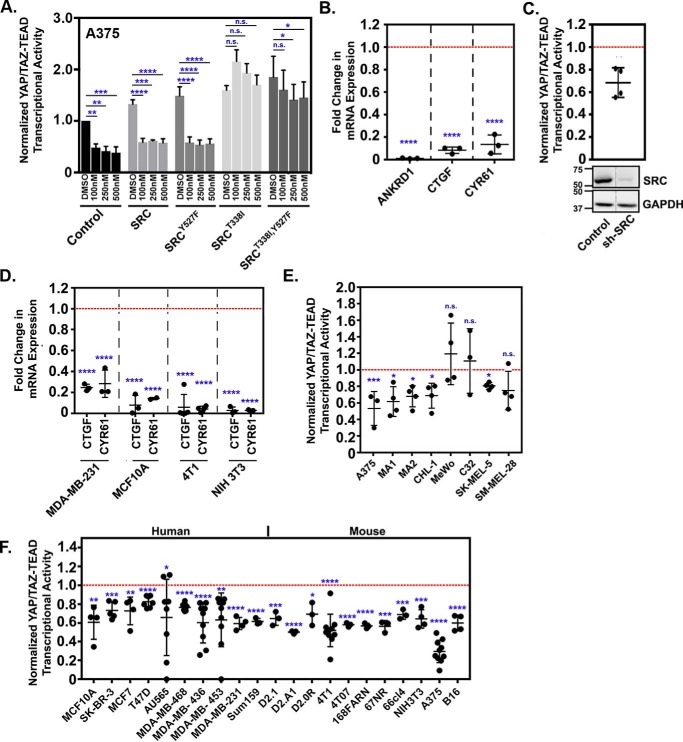
**SRC inhibition reduces YAP/TAZ transcriptional activity and target gene expression.** *A*, A375 cells stably expressing control empty vector, WT SRC, SRC^Y527F^, or dasatinib-resistant forms of WT SRC (SRC^T338I^) or SRC^Y527F^ (SRC^Y527F,T338I^) were assayed for YAP/TAZ-TEAD transcriptional activity following 6.5-h treatment with the indicated dose of dasatinib (*n* = 3 independent experiments). *B*, A735 cells were treated with DMSO or 500 nm dasatinib for 6.5 h and then assayed by qPCR. *C*, A735 cells stably expressing either a control or an SRC shRNA (pRetroSuper-shSRC) were assayed by Western blotting and for YAP/TAZ-TEAD transcriptional activity. Note that both lanes were from the same blot but were not adjacent. *D–F*, the indicated cell lines were treated with DMSO, 500 nm dasatinib (*D* and *F*), or 100 nm dasatinib (*E*) for 6.5 h and then assayed by qPCR (*D*) or for YAP/TAZ-TEAD transcriptional activity (*E* and *F*). Scatter plots show mean ± S.D. (*error bars*), where each *dot* is an independent experiment in which the dasatinib-treated cells (*B* and *D–F*) or SRC shRNA cells (*C*) were converted to -fold change from control cells, which are indicated by the *red dotted line*. Statistical significance was tested using two-way ANOVA with Dunnett's multiple-comparison test (*A*) and Student's *t* test (*B–F*). *n.s.*, *p* > 0.05; *, *p* ≤ 0.05; **, *p* ≤ 0.01; ***, *p* ≤ 0.001; ****, *p* ≤ 0.0001.

We next tested whether SRC was required for YAP/TAZ transcriptional activity and target gene expression. Inhibition of SRC family kinases (SFKs) via dasatinib treatment decreased YAP/TAZ transcriptional activity in a dose-dependent manner in human melanoma cells (A375), mouse mammary carcinoma cells (4T1), and mouse fibroblasts (NIH3T3) (Fig. S1, *A* and *B*) at both high and low density. The inhibition was greater at low density, likely because YAP/TAZ activity is already reduced when cells are cultured at high densities (Fig. S1*A*). To ensure that inhibition of YAP and TAZ by dasatinib was due to inhibition of SRC, we performed a rescue experiment, where A375 cells stably expressing control empty vector, WT SRC, activated SRC^Y527F^, or dasatinib-resistant forms of SRC (SRC^T338I^) or SRC^Y527F^ (SRC^T338I,Y527F^) were treated with dasatinib and assayed for YAP/TAZ transcriptional activity. All doses of dasatinib that we tested impaired YAP/TAZ transcriptional activity in cells expressing control empty vector, WT SRC, or activated SRC^Y527F^. In contrast, cells expressing the dasatinib-resistant forms of SRC or SRC^Y527F^ maintained high YAP/TAZ activity even at the highest dose of dasatinib (Fig. 2*A*). Representative Western blots confirmed expression of each mutant SRC construct and that dasatinib effectively reduced the levels of phosphorylated SRC family kinases (Fig. S1*C*). YAP/TAZ transcriptional activity was also reduced following partial knockdown of SRC (Fig. 2*C*) or by treatment with two other SFK inhibitors, PP2 and saracatinib (data not shown). SRC inhibition also reduced the expression of known YAP/TAZ-regulated genes (*CTGF*, *CYR61*, and ankyrin repeat domain 1 (*ANKRD1*)) (Fig. 2, *B* and *D*). SRC inhibition reduced YAP/TAZ activity in the majority (25 of 28) of human and mouse melanoma and breast cancer cell lines that we tested (Fig. 2, *E* and *F*). Interestingly, two of the lines that maintained YAP/TAZ activity following dasatinib treatment, MeWo and C32, also showed the least dramatic decrease in pSFK following dasatinib treatment (Fig. S2*B*). In addition, these lines showed an increase in YAP/TAZ activity following transfection with SRC^Y527F^ (Fig. 1*B*). This suggests that SRC does influence YAP/TAZ in these cells but that they are more resistant to dasatinib. Together, these results show that SRC is required for YAP/TAZ activity and target gene expression in many breast cancer and melanoma cell lines.

### SRC promotes YAP/TAZ activity by repressing LATS-mediated phosphorylation of YAP and TAZ

**Figure 3.**
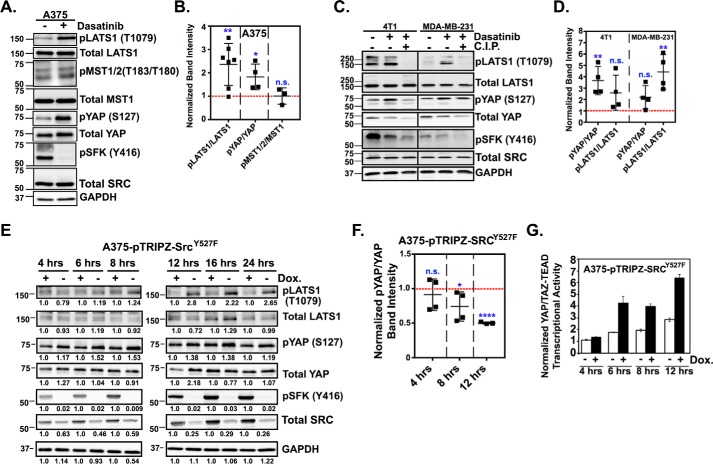
**SRC represses LATS activation and inhibitory phosphorylation of YAP and TAZ.** *A* and *C*, representative experiments in which the indicated cells were treated with DMSO or dasatinib for 1 h and then assayed by Western blotting. Lysates from dasatinib-treated cells were treated with calf intestinal phosphatase (*C.I.P*) and run in parallel to ensure that the detected bands were phosphorylated proteins. *B* and *D*, band intensities for total and phosphorylated YAP, LATS1, and MST were quantified, normalized to GAPDH, and plotted as scatter plots showing mean ± S.D. (*error bars*). *E* and *F*, A375 cells stably expressing a doxycycline-inducible form of SRC^Y527F^ were treated with 0.5 μg/ml doxycycline for the indicated times and then assayed by Western blotting. *E*, one representative experiment with relative band intensities indicated. *F*, the average ratio of phosphorylated over total YAP, LATS, and MST for all experiments. *G*, cells from *E* were assayed for YAP/TAZ-TEAD transcriptional activity (*n* = 4 replicates from one experiment). In scatter plots, each *dot* is an independent experiment in which the dasatinib-treated cells (*B* and *D*) or doxycycline-treated cells (*F*) were converted to -fold change over control cells, which are indicated by the *red dotted line*. Statistical significance was tested using Student's *t* test. *n.s.* = *p* > 0.05; *, *p* ≤ 0.05; **, *p* ≤ 0.01; ****, *p* ≤ 0.0001.

Regulation of YAP and TAZ can occur through both Hippo pathway-dependent and -independent mechanisms, so we next tested whether altering SRC activity influenced the Hippo pathway. Dasatinib treatment of A375 cells significantly increased the phosphorylation of serine 127 and serine 397 of YAP and serine 89 of TAZ, all of which are key LATS-inhibitory phosphorylation sites (Fig. S2 and Fig. 3, *A* and *B*). A time course revealed that YAP and TAZ phosphorylation increased within 15 min of dasatinib treatment and remained high for at least 6 h (Fig. S2*A*). A similar dasatinib-mediated increase in YAP phosphorylation was also observed in several additional cell lines (Fig. 3, *C* and *D*, and Fig. S2, *B* and *C*). Dasatinib treatment also significantly increased the levels of phosphorylated (*i.e.* activated) LATS in multiple cell lines but did not alter MST phosphorylation (Fig. 3, *A–D*, and Fig. S2, *A* and *C*). The increased intensity of the pLATS and pYAP bands in the dasatinib-treated samples was significantly reduced if the sample was incubated with calf intestinal phosphatase (*C.I.P.*) prior to Western blotting, confirming that the bands are phosphorylated proteins (Fig. 3*C*). A similar dasatinib-mediated increase in pLATS was also observed if we immunoprecipitated endogenous LATS and blotted for pLATS (Fig. S2*D*). PP2 and saracatinib also each promoted YAP and LATS phosphorylation (data not shown). These data strongly suggest that loss of SRC activity leads to LATS activation and subsequent inhibitory phosphorylation of YAP and TAZ. However, not all of the cell lines that showed reduced YAP/TAZ activity following dasatinib treatment (Fig. 2, *E* and *F*) also showed reduced inhibitory phosphorylation of YAP (Fig. S2*B*), suggesting that in some cells, SRC regulates YAP and TAZ through a different, LATS-independent, mechanism (see “Discussion”).

We next tested whether SRC activation represses LATS and reduces YAP phosphorylation. For this, we generated A375 cells stably expressing doxycycline-inducible SRC^Y527F^. Dose–response experiments revealed that 0.5 μg/ml doxycycline was sufficient to induce a modest increase in the expression of SRC^Y527F^, which significantly increased SRC activation (data not shown). Time-course experiments showed a significant activation of SRC within 8 h of doxycycline treatment, and this corresponded with a decrease in LATS and YAP phosphorylation (Fig. 3, *E* and *F*) and a significant increase in YAP/TAZ transcriptional activity (Fig. 3*G*). These results suggest that SRC activates YAP and TAZ by inhibiting LATS.

**Figure 4.**
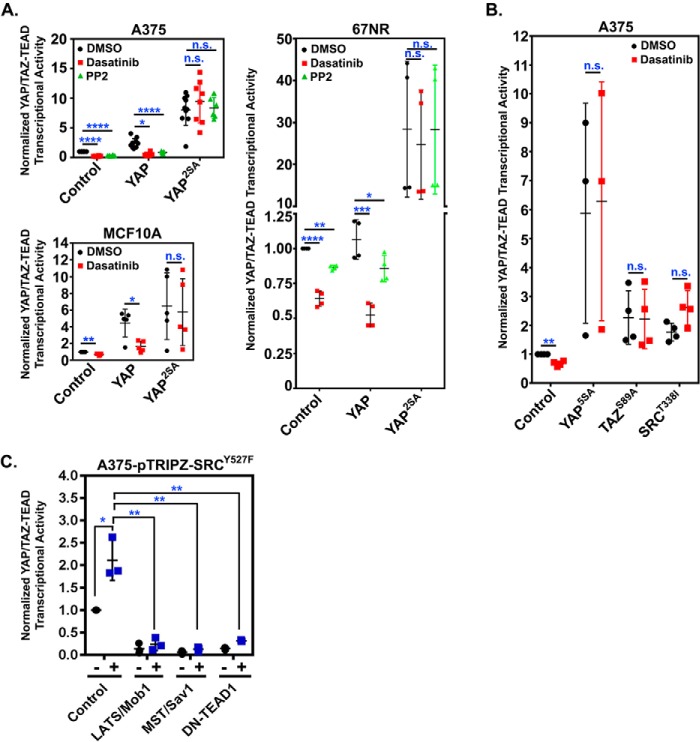
**LATS-insensitive YAP and TAZ are resistant to SRC inhibition.** *A*, the indicated cell lines stably expressing control empty vector, WT YAP (*YAP*), or LATS-insensitive YAP (*YAP^2SA^*) were treated with DMSO, dasatinib (500 nm), or PP2 (10 μm) for 6.5 h and then assayed for YAP/TAZ-TEAD transcriptional activity. *B*, A375 cells were transiently transfected overnight with control empty vector, dasatinib-resistant SRC (SRC^T388I^), or LATS-insensitive YAP (YAP^5SA^) or TAZ (TAZ^S89A^) and YAP/TAZ-TEAD reporter constructs. Cells were then treated for 6.5 h with DMSO or dasatinib (500 nm) and assayed for YAP/TAZ-TEAD transcriptional activity. *C*, A375 cells stably expressing a doxycycline-inducible form of SRC^Y527F^ were transiently transfected overnight with the indicated vectors and YAP/TAZ-TEAD reporter constructs and then treated for 12 h with 0.5 μg/ml doxycycline and assayed YAP/TAZ-TEAD transcriptional activity. Scatter plots show mean ± S.D. (*error bars*), where each *dot* is an independent experiment. Statistical significance was tested using Student's *t* test with Bonferroni correction for multiple comparisons. *n.s.*, *p* > 0.05; *, *p* ≤ 0.05; **, *p* ≤ 0.01; ***, *p* ≤ 0.001; ****, *p* ≤ 0.0001.

If SRC indeed acts on YAP and TAZ by repressing LATS, then the activity of LATS-insensitive YAP or TAZ mutants should not be dependent upon SRC. To test this possibility, cells stably expressing control empty vector, WT YAP, or a YAP mutant in which the two key LATS phosphorylation sites (Ser-127 and Ser-397) are mutated from serine to alanine (YAP^2SA^) were treated with PP2 or dasatinib and assayed for YAP/TAZ transcriptional activity. Dasatinib treatment significantly reduced YAP/TAZ activity in cells expressing the control vector and WT YAP, but not in cells expressing the LATS-insensitive YAP^2SA^ (Fig. 4*A*). Similar results were obtained in cells transiently transfected with another LATS-insensitive YAP mutant YAP^5SA^, which cannot be phosphorylated on any of the known LATS phosphorylation sites (Fig. 4*B*) or with a mutant TAZ (TAZ^S89A^) that lacks a key LATS phosphorylation site (Fig. 4*B*). Conversely, activation of LATS by overexpression of either MST1/Sav or LATS1/Mob1 repressed SRC^Y527F^-mediated YAP/TAZ activation (Fig. 4*C*). Collectively, these results indicate that SRC activation promotes YAP/TAZ activity by repressing LATS.

### Adhesion-mediated activation of endogenous SRC represses LATS and promotes YAP/TAZ activity

**Figure 5.**
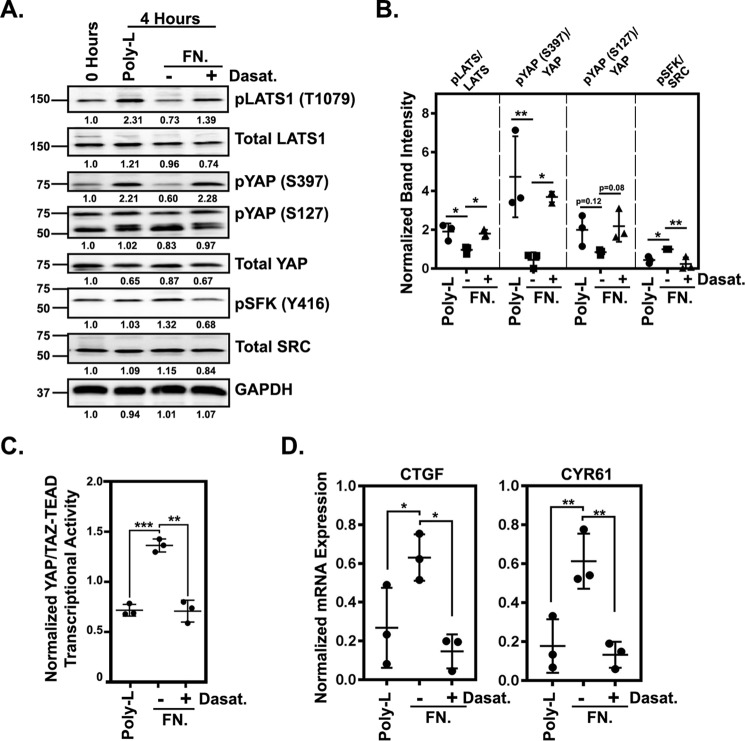
**Cell-ECM adhesion promotes YAP/TAZ activity in an SRC-dependent manner.** *A*, a representative set of Western blots from A375 cells following adhesion assays (see “Experimental procedures”) in which the cells were plated on fibronectin (*FN*) or poly-l-lysine for 1 h and then treated with DMSO or 500 nm dasatinib and cultured for an additional 3 h. Band intensities, normalized to the sample collected at 0 h, are indicated *below* each blot. *B*, quantification of three separate experiments performed as in *A*. For each, band intensities for the fibronectin with and without dasatinib and poly-l-lysine samples were normalized to the 0-h sample and then to GAPDH. Then the ratio of phosphorylated to total protein was calculated and plotted. *C* and *D*, adhesion assays with A375 cells plated on fibronectin or poly-l-lysine for 1 h and then treated with DMSO or 500 nm dasatinib and cultured for an additional 6.5 h before being assayed for YAP/TAZ-TEAD transcriptional activity (*C*) or by qPCR (*D*). All samples in *C* and *D* were normalized to a starting sample collected at 0 h. Scatter plots show mean ± S.D. (*error bars*), where each *dot* is an independent experiment. Statistical significance was tested using one-way ANOVA with Dunnett's multiple-comparison test (*B* and *D*) and Student's *t* test with Bonferroni correction for multiple comparisons (*C*). *, *p* ≤ 0.05; **, *p* ≤ 0.01; ***, *p* ≤ 0.001.

The SRC^Y527F^ oncogene is not a common driver mutation in cancer. Instead, the elevated SRC activity observed in many human cancers is the result of aberrant signaling by other pathways. Therefore, we next tested whether activation of endogenous SRC promotes YAP/TAZ activity through inhibition of LATS. A major driver of SRC activation is integrin-mediated adhesion to the extracellular matrix. Consistently, we found that melanoma cells attached and spreading on either fibronectin or collagen had significantly higher levels of activated SRC than cells adhering in an integrin-independent manner to poly-l-lysine (Fig. 5, *A* and *B*, and Fig. S3, *A* and *B*). Adhesion to fibronectin also reduced YAP and LATS phosphorylation (Fig. 5, *A* and *B*, and Fig. S3, *A* and *B*) and increased YAP/TAZ transcriptional activity (Fig. 5*C* and Fig. S3*C*) as well as CTGF and CYR61 mRNA expression (Fig. 5*D* and Fig. S3*D*). Dasatinib treatment prevented the adhesion-mediated decrease in LATS and YAP phosphorylation (Fig. 5, *A* and *B*) and the increase in YAP/TAZ activity and CTGF and CYR61 mRNA expression (Fig. 5, *C* and *D*), indicating that these effects are SRC-dependent. These results show that activation of endogenous SRC through integrin–ECM adhesion represses LATS activity and promotes YAP/TAZ function.

### SRC prevents GIT1/LATS-mediated repression of YAP and TAZ

As described in our recent review (22), several recent publications have identified SRC-mediated activation of YAP/TAZ in a variety of cell types. Consistent with our results, several of these studies show that SRC can repress LATS-mediated phosphorylation of YAP and TAZ. However, several distinct SRC-dependent mechanisms have been described, including pathways involving PI3K and phosphoinositide-dependent kinase 1 (PDK1) (47, 48), Rho (49), or c-Jun N-terminal kinase (JNK) (51). SRC can also directly phosphorylate LATS (50). We performed several experiments to test the role of each of these mechanisms in melanoma cells and found that none was sufficient to explain how SRC was regulating LATS in these cells (Fig. S4). Briefly, neither JNK nor PI3K inhibition reduced basal YAP/TAZ activity (Fig. S4*A*). PI3K inhibition was also unable to prevent the SRC^Y527F^-mediated increase in YAP/TAZ activity (Fig. S4, *A* and *B*), and activated PI3K expression could not rescue cells from dasatinib-mediated repression of YAP/TAZ activity (Fig. S4*C*). We also found no evidence of SRC-mediated tyrosine phosphorylation of LATS in A375 cells (Fig. S4*D*). Expression of either activated RhoA or activated RhoC could rescue YAP/TAZ activity in cells treated with dasatinib, but neither was able to reduce the LATS-mediated phosphorylation of YAP (Fig. S4*E*), suggesting that Rho is promoting YAP/TAZ activity through a different pathway than SRC. Consistently, whereas expression of dominant-negative RhoA or RhoC constructs did reduce YAP/TAZ activity in both control and SRC^Y527F^-expressing cells (Fig. S4*F*), SRC activation still significantly increased YAP/TAZ activity in cells expressing these dominant-negative Rho constructs (Fig. S4*F*). Collectively, these data suggest that another SRC effector pathway that regulates LATS must exist.

**Figure 6.**
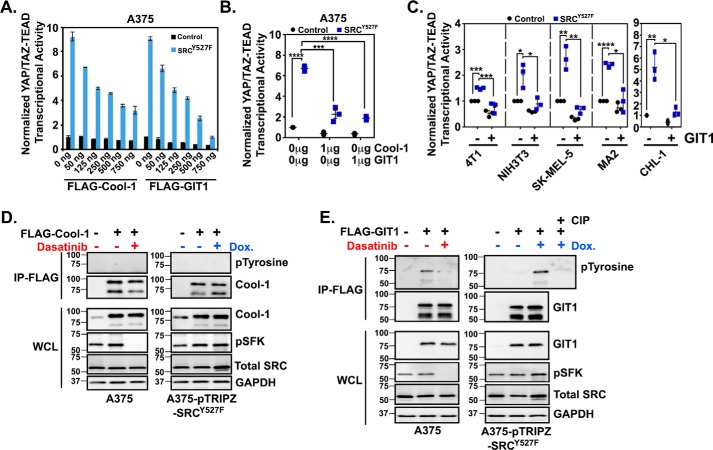
**GIT1 is an SRC effector protein that regulates YAP/TAZ activity.** *A–C*, the indicated cell lines were co-transfected with either control empty vector or SRC^Y527F^ and GIT1 or Cool-1 DNA and then assayed for YAP/TAZ-TEAD transcriptional activity 24 h later. *D* and *E*, A375 or A375-pTRIPZ-SRC^Y527F^ cells were transfected with FLAG-tagged GIT1 or FLAG-tagged Cool-1. After 24 h, A375 cells were treated with either DMSO (−) or 500 nm dasatinib (+) for 1 h (*left*), and A375-pTRIPZ-SRC^Y527F^ cells were treated with water (−) or 0.5 μg/ml doxycycline (+) for 12 h (*right*). Western blots were then performed on whole-cell lysate (*WCL*) or following IP with an anti-FLAG antibody for phosphorylated tyrosine, GIT1, Cool-1 pSFK, or GAPDH. As a control, the indicated sample was also incubated with calf intestinal phosphatase (*CIP*) prior to loading on the gel. *A*, *n* = 4 (one experiment performed in quadruplicate). Scatter plots show mean ± S.D. (*error bars*), where each *dot* is an independent experiment. Statistical significance was tested using Student's *t* test with Bonferroni correction for multiple comparisons. *, *p* ≤ 0.05; **, *p* ≤ 0.01; ***, *p* ≤ 0.001; ****, *p* ≤ 0.0001.

Because we saw no influence of SRC on MST phosphorylation, we looked for proteins that regulate LATS that are also potential SRC effectors. Cool-1 (ARHGEF7/β-PIX) and GIT1 are both regulated by SRC phosphorylation (52–55), and each can promote Hippo pathway–dependent repression of YAP and TAZ (56, 57). We found that overexpression of either Cool-1 or GIT1 repressed SRC-mediated YAP/TAZ activity in a dose-dependent manner (Fig. 6, *A* and *B*). GIT1 could also repress SRC-mediated YAP/TAZ activity in several additional cell lines (Fig. 6*C*). We next tested whether GIT1 or Cool-1 phosphorylation was SRC-dependent in our cells. Although we found no evidence of Cool-1 phosphorylation (Fig. 6*D*), tyrosine phosphorylation of GIT1 was increased by SRC activation and decreased by SRC inhibition (Fig. 6*E*). These results identify GIT1 as another SRC effector that influences YAP/TAZ activity in breast cancer and melanoma cells.

### SRC-mediated YAP/TAZ activation promotes tumor growth and metastasis

**Figure 7.**
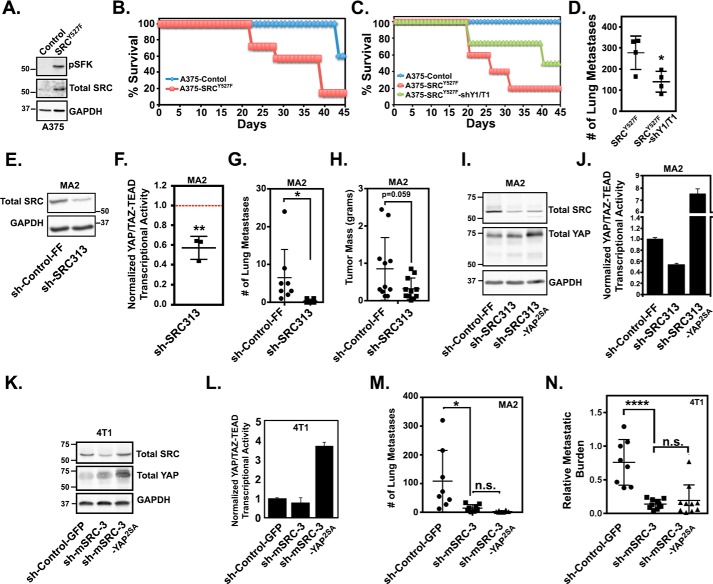
**SRC-mediated YAP/TAZ activation promotes tumor growth and metastasis.** *A*, A375 cells stably expressing control empty vector or SRC^Y527F^ were assayed by Western blotting. *B*, 5 × 10^5^ cells from *A* were injected subcutaneously into NOD/Scid mice, and mouse survival was assayed as described under “Experimental procedures.” *C*, A375 cells expressing a control empty vector, SRC^Y527F^, or SRC^Y527F^ and tandem YAP and TAZ shRNAs (shY1/T1) were injected subcutaneously into NOD/Scid mice to assay mouse survival. *D*, cells from *C* were also injected into the lateral tail vein of NSG mice, and lung metastases were counted after 21 days. *E* and *F*, MA2 cells stably expressing a control shRNA (sh-Control-FF) or an SRC shRNA (sh-SRC313) were assayed by Western blotting (*E*) or for YAP/TAZ-TEAD transcriptional activity (*F*). The scatter plot (*F*) shows mean ± S.D. (*error bars*), and each *dot* is an independent experiment in which sh-SRC313 cells were converted to -fold change from control cells (*red dotted line*). *G* and *H*, 5 × 10^5^ cells from *E* were injected either into the lateral the tail vein (*G*) or subcutaneously (*H*) into NOD/Scid mice, and after 5 weeks, lung metastases were counted (*G*), and primary tumors were isolated and weighed (*H*). *I–L*, MA2 or 4T1 cells stably expressing control shRNAs (sh-Control-FF or sh-Control-GFP), SRC shRNAs (sh-SRC313 and sh-mSRC-3), or SRC shRNAs and LATS-insensitive YAP^2SA^ were assayed by Western blotting (*I* and *K*) or for YAP/TAZ-TEAD transcriptional activity (*J* and *L*). *M*, 1 × 10^6^ of the MA2 cells from *I* were injected into the lateral tail veins of NOD/Scid mice, and after 5 weeks, lung metastases were counted. *N*, 2.5 × 10^4^ of the 4T1 cells from *K* were injected into the lateral tail veins of BALB/c mice, and after 2 weeks, lung metastatic burden was quantified using qPCR. Scatter plots in *D*, *G*, *H*, *M*, and *N* show mean ± S.D., where each *dot* is a mouse. The plot in *F* shows mean ± S.D., where each *dot* is an independent experiment in which the SRC shRNA cells were converted to -fold change from control cells (*red dotted line*). Statistical significance was tested using Student's *t* test (*D*, *F*, *G*, and *H*) and one-way ANOVA with Dunnett's multiple-comparison test (*M* and *N*). *n.s.*, *p* > 0.05; *, *p* ≤ 0.05; ****, *p* ≤ 0.0001.

Like YAP and TAZ, SRC is a known driver of cancer progression and metastasis (33–36, 39–41, 58). Therefore, we next sought to determine whether SRC-dependent activation of YAP and TAZ is important for tumor growth and metastases. First, we tested whether SRC-mediated YAP/TAZ activation is sufficient to promote tumor growth and metastasis. Compared with control A375 cells, A375 cells stably expressing activated SRC^Y527F^ formed tumors that grew significantly faster and required the mice to be euthanized much sooner (Table S1 and Fig. 7, *A* and *B*). Partial knockdown of both YAP and TAZ, through stable expression of tandem YAP and TAZ miR30-based shRNAs, significantly reduced this SRC-mediated tumor growth and extended mouse survival (Table S1 and Fig. 7*C*) and also significantly reduced metastasis formation (Fig. 7*D*). A second independent experiment that included an additional tandem YAP/TAZ shRNA construct gave similar results (Fig. S5, *A–D*). These findings show that YAP/TAZ activity is required for SRC-mediated tumor growth and metastasis.

Next, we tested whether inhibition of SRC prevents tumor growth and metastasis. Partial SRC knockdown in metastatic human melanoma cells (MA2) significantly inhibited YAP/TAZ transcriptional activity and reduced the number of metastases that formed (Fig. 7, *E–G*). SRC knockdown also led to a roughly 50% reduction in primary tumor size (Fig. 7*H*), but this difference was not quite statistically significant. Thus, inhibition of SRC, which significantly decreases YAP/TAZ activity, reduces tumor growth and metastasis.

Next, we tested whether restoring YAP activity could rescue metastasis in the SRC knockdown cells. Stable expression of the LATS-insensitive YAP^2SA^ in the metastatic melanoma cells with SRC knockdown was unable to rescue metastasis, despite significantly enhancing YAP/TAZ activity (Fig. 7, *I*, *J*, and *M*). Similar results were obtained in a second independent experiment (Fig. S5, *E* and *F*). To test whether this finding was cancer-type specific, we repeated this experiment with metastatic breast cancer cells (4T1). As we observed in the melanoma cells, knockdown of SRC significantly inhibited YAP/TAZ activity and reduced metastatic burden in these breast cancer cells (Fig. 7, *K*, *L*, and *N*). Once again, stable expression of the LATS-insensitive YAP^2SA^ was unable to rescue metastasis, despite significantly enhancing YAP/TAZ activity (Fig. 7, *K*, *L*, and *N*). These findings suggest that although one critically important pro-metastatic function of SRC is to drive YAP/TAZ activity, SRC must also promote other YAP/TAZ-independent pathways essential for metastasis. Nevertheless, our results indicate that SRC-mediated YAP/TAZ activation drives tumor growth and metastasis and suggest that this pathway is a potential therapeutic target.

## Discussion

### SRC-mediated YAP/TAZ activation as a therapeutic target in cancer

YAP and TAZ are known drivers of tumor formation, progression, and metastasis in many cancer types, including breast cancer and melanoma, and experimental models suggest that preventing aberrant YAP/TAZ activation can inhibit tumor growth and prevent metastasis (22–24). This has led to great enthusiasm for YAP/TAZ as therapeutic targets. However, given that YAP and TAZ have important functions in normal tissues, systemic inhibition in cancer patients seems likely to result in adverse side effects. The identification of cancer-associated pathways that promote aberrant YAP/TAZ activity will enable a more targeted treatment strategy. Our results show that inhibition of SRC represses YAP/TAZ transcriptional activity in the majority of human melanoma and breast cancer cell lines that we tested. Although SRC is rarely mutated in human cancers, its activity is frequently elevated. Our findings suggest that this elevated SRC activity may be one important driver of the aberrantly high YAP/TAZ activity observed in many human cancers. Consistently, we found that blocking SRC reduced YAP/TAZ activity and impaired tumor growth and metastasis. Reciprocally, our results also show that YAP/TAZ activation is critically important downstream of the long-established oncogene, SRC, and link these separately discovered, powerful drivers of tumor progression.

Our previous results show that, like SRC, YAP activation can promote metastasis in A375 and 4T1 cells (25). However, SRC was present in these cells, and our current finding that YAP activation is not sufficient for metastasis in the absence of SRC indicates that both SRC and YAP/TAZ activation are essential for metastasis. This suggests that SRC drives metastasis in a manner that requires YAP and TAZ, but also that SRC promotes other YAP/TAZ-independent pathways important for metastasis. This notion is not surprising, given the wealth of data linking SRC to pro-metastatic pathways (33–42). Nevertheless, our data show that SRC is essential to maintain YAP/TAZ activity in many breast cancer and melanoma cells and that elevated SRC-mediated YAP/TAZ activity is required for metastasis. This makes therapies that target SRC a potential treatment for YAP/TAZ-dependent cancers. Notably, Food and Drug Administration–approved therapies that target SRC already exist and could potentially be repurposed for use in cancers in which SRC is driving YAP/TAZ activation.

The fact that SRC is required for YAP/TAZ activity in so many cell lines (25 of 28) suggests that this is an important and relevant regulatory pathway in these cancer types rather than a pathway unique to a few cell lines. However, three cell lines we tested did not require SRC for YAP/TAZ activity (Fig. 2), and some others showed only a modest decrease in YAP/TAZ activity when SRC was inhibited. This suggests that SRC is not the only cause of YAP/TAZ activation in cancer. The existence of other SRC-independent mechanisms means that diagnostic tools that can identify tumors dependent upon SRC for YAP/TAZ-mediated tumorigenesis and metastasis are essential. As such, it is worth noting that immunohistochemical analysis can determine the levels of phosphorylated SRC and nuclear YAP/TAZ in human tumor biopsies. It will be important to determine whether this is a reliable way to identify cancers dependent upon this pathway.

### GIT1 is an SRC effector that regulates YAP and TAZ

We revealed a new SRC effector protein that regulates YAP and TAZ in breast cancer and melanoma cells. Consistent with studies in nontransformed cells (52, 55), we found that GIT1 is phosphorylated in an SRC-dependent manner in cancer cells. GIT1 repressed SRC-mediated YAP/TAZ activity in each of the six cell lines we tested (Fig. 6, *B* and *C*), suggesting that this regulatory mechanism is not unique to one cell line or cancer type.

How SRC-mediated phosphorylation of GIT1 represses LATS is not yet clear. A previous study found that the fly homologs of GIT1 and Cool-1 collaborate as scaffolds to promote Hippo activation (57), and another study showed that Cool-1 represses YAP and TAZ by scaffolding them with LATS (56). Cool-1 binds GIT1, and this interaction is regulated by SRC-mediated phosphorylation of Cool-1 (59–61). Although we found that Cool-1 overexpression can also repress SRC-mediated YAP/TAZ activation (Fig. 6*A*), we found no evidence that SRC influenced Cool-1 phosphorylation in these cells (Fig. 6*D*). Therefore, although Cool-1 may be involved, it does not appear to be the SRC effector in this pathway. Perhaps GIT1 and Cool-1 interact and enhance the formation of the Hippo pathway complex that represses YAP and TAZ, and SRC-mediated phosphorylation of GIT1 dissociates this complex. Alternatively, phosphorylation of GIT1 by SRC may alter its localization in the cell, preventing it from activating LATS. Indeed, both GIT1 and Cool-1 localize to focal adhesions, where they are regulated by focal adhesion kinase (FAK)/SRC signaling (55, 60, 62), but whether this influences LATS was not explored.

Paradoxically, another study found that GIT1 and Cool-1 enhance focal adhesion signaling to increase YAP/TAZ activity (63). This may suggest that GIT1 and Cool-1 can act to either repress or activate YAP and TAZ, depending on where they localize in the cell. GIT1 and Cool-1 also bind to Scribble (64), an important upstream regulator of the Hippo pathway. Therefore, a third possibility is that SRC regulates this interaction to influence LATS activity. An important future step in deciphering these complicated signaling events is to identify the SRC-dependent phosphorylation site(s) on GIT1 and determine how they impact GIT1 and LATS function.

### SRC can influence YAP and TAZ through multiple distinct mechanisms

**Figure 8.**
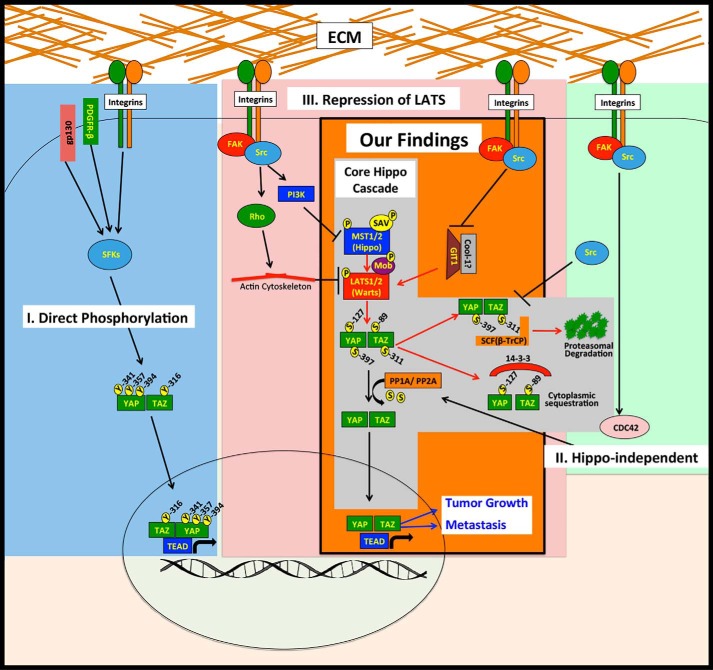
**SRC promotes YAP/TAZ activity through multiple downstream pathways.** Shown is a model that summarizes our findings in the context of what has been previously reported about SRC regulation of YAP/TAZ. Our findings (*orange box*) show that SRC-mediated repression of LATS promotes YAP/TAZ activity and drives tumor growth and metastasis and that GIT1 is an important SRC effector that regulates LATS. The *gray box* indicates the core Hippo kinase cascade. The other known mechanisms of SRC regulation of YAP and TAZ include direct phosphorylation (*blue box*) (*I*), Hippo-independent mechanisms (*green box*) (*II*), and activation of pathways that repress LATS (*red box*) (*III*).

Although GIT1 can influence SRC-mediated YAP/TAZ activity in multiple cell lines (Fig. 6), we did not test its role in every cell line used in this study. Nor did we rule out a role for other SRC effector pathways in each cell line. Thus, whereas GIT1 is clearly one SRC effector that regulates YAP and TAZ, it is likely that other SRC effectors are also important. Consistently, several recent papers have described roles for SRC and SRC-family kinases in the regulation of YAP/TAZ activity, and our recent review thoroughly discusses these papers (22). Interestingly, the mechanisms downstream of SRC that have been reported to influence YAP and TAZ vary greatly. These fall into three distinct categories (Fig. 8, *I–III*). Several studies show that SRC and other SRC family kinases can directly phosphorylate YAP (65–70) or TAZ (71) to promote their protein stability, transcriptional activity, and/or interaction with other transcription factors (Fig. 8, *I*). SRC can also influence YAP and TAZ through distinct Hippo pathway-independent mechanisms (Fig. 8, *II*) (72, 73). Finally, SRC can activate YAP and TAZ by repressing LATS (47–50, 74, 75) (Fig. 8, *III*). Consistent with these latter studies, we found that in breast cancer and melanoma cells, SRC can promote YAP/TAZ activity by repressing LATS (Fig. 8, our findings). However, known SRC effectors that were shown by others to influence LATS, such as PI3K, JNK, and Rho, did not seem to play a major role in the cells we tested (Fig. S4). Thus, whereas multiple SRC effector pathways that can influence YAP/TAZ activity clearly exist, which of these pathways is active appears to be cell type– and context–dependent. Consistently, not all of the cell lines that we found to have reduced YAP/TAZ activity following SRC inhibition also showed increased YAP phosphorylation, indicating that, in these cells, SRC is influencing YAP and TAZ through LATS-independent mechanisms. This suggests that blocking individual SRC effector pathways may not always effectively inhibit SRC-mediated YAP/TAZ activity because other SRC effector pathways could compensate. However, because SRC influences several distinct YAP/TAZ activation pathways, direct inhibition of SRC itself may be a very good therapeutic strategy, if used in the right patients.

### Cell-ECM adhesion promotes SRC-mediated YAP/TAZ activity

Numerous oncogenic pathways activate SRC, and many signals from the tissue microenvironment that are altered in cancer can drive SRC activation (76–79). Another intriguing question that emerges from our work is which of these mechanisms of SRC activation promotes YAP/TAZ transcriptional activity. Several recent papers identified integrins that regulate YAP and TAZ (reviewed in Ref. 22), and SRC appears to be essential for YAP/TAZ activation by several integrins in both normal and cancerous cells (47–49, 65, 74, 75, 80). Consistently, we found that integrin-mediated adhesion to fibronectin repressed LATS-mediated phosphorylation of YAP and promoted YAP/TAZ function in an SRC-dependent manner (Fig. 5). This suggests that integrins are one important driver of SRC-mediated YAP/TAZ activity in cancer cells.

Several other mechanisms of SRC activation have also been found to drive YAP/TAZ activation. Interleukin-6/gp130 (68), platelet-derived growth factor receptor (67), and the polyomavirus middle T-antigen oncogene can each activate SRC to increase YAP/TAZ activity (72, 81). In addition, many of the same cancer-associated microenvironmental cues and oncogenic events that regulate SRC also influence YAP and TAZ. Indeed, changes in growth factor signaling, cell–cell adhesion, G-protein–coupled receptor signaling, metabolic cues, and cell polarity cues have each been shown to increase SRC activity in cancer cells (76–79, 82), and each of these can also increase YAP/TAZ activity (18, 19, 21, 83). It will be interesting to determine whether any of these drivers of SRC activation also lead to aberrant YAP/TAZ activity and, if so, whether blocking these cues can prevent YAP/TAZ-mediated tumor growth and metastasis.

In summary, we show that SRC is critical for YAP/TAZ activity in many breast cancer and melanoma cell lines and that SRC-mediated activation of YAP and TAZ plays important roles in tumor growth and metastasis. This makes SRC, and the pathways that activate it, potential therapeutic targets in cancers that are dependent upon YAP and TAZ.

## Experimental procedures

### Cell lines, vectors, and cloning

Information about the cell lines used, culture conditions, and their source are listed in Table S2. All cells were cultured at 37 °C and 5% CO_2_ and were maintained at low passage number. Cell lines were routinely tested for mycoplasma and other bacterial contaminants. All vectors used in this work are listed in Table S3. If vectors were previously described, received as gifts, or purchased from commercial vendors, we listed the source of the vector. New vectors were generated using standard cloning procedures, and the source constructs used for each insert and each vector backbone are indicated in Table S3. For some RNAi, we either used commercial constructs (GE Dharmacon) or built our own. For the latter, miR30-based shRNAs were designed, synthesized as 97-mers (Integrated DNA Technologies), and then cloned into the miR30 backbone located in the 3′-UTR of the *pac* gene (puromycin resistance) in the MSCV-ZSG-2A-Puro-miR30 vector, as described previously (25). For tandem YAP/TAZ shRNAs, miR30-based shRNAs targeting YAP and TAZ were cloned in tandem into the MSCV-ZSG-2A-Puro-miR30 vector. shRNA 97-mer sequences are listed in Table S4. All newly developed vectors were confirmed by test restriction enzyme cuts and sequenced to confirm their identity.

### Generation of retrovirus and lentivirus

Retrovirus and lentivirus were packaged as follows. 293FT cells were plated on 6-well plates at roughly 50% confluence in full-growth medium and 16–24 h later were transfected with a mixture of 1 μg of viral vector, 0.5 μg of packaging vector (pCL-eco or gag/pol for retrovirus, psPAX2 for lentivirus), 0.5 μg of coat protein (VSVG or pHCMV-EcoEnv), 5 μl of X-tremeGENE^TM^ 9 (Sigma), and 95 μl of Opti-MEM^TM^ (Gibco). The transfection mix was prepared using the manufacturer's protocol and added to the cells for 16–24 h, after which the mix was removed and the cells were fed with fresh full-growth medium. Cells were then cultured for an additional 24 h, after which the culture supernatant was collected and filtered through a 0.45-μm filter and then either stored at −80 °C or immediately used. For stable transduction, cells were plated at roughly 50% confluence, and then, 16–24 h later, viral supernatant diluted 1:1 with fresh growth medium was added to the cells. Polybrene (Sigma) was also added at 8 μg/ml. After 24 h, viral supernatants were removed, and cells were fed with fresh growth medium and then stably selected with the appropriate antibiotic.

### YAP/TAZ transcriptional activity

The YAP/TAZ transcriptional reporter assays utilized a YAP/TAZ reporter construct (pGL3–5xMCAT(SV)-49 (84)) that consists of five repeats of a TEAD-responsive promoter driving expression of the firefly luciferase gene and a control *Renilla* luciferase construct PRL-TK (Promega). Typically for these assays, cells were plated on 12-well dishes in duplicate and co-transfected with 400 ng of a 20:1 mixture of pGL3-5xMCAT(SV)-49 and PRL-TK using Lipofectamine Plus (Invitrogen). After 24 h, luciferase activity was assayed using the Dual-Luciferase Reporter Assay System (Promega) as described previously (25). For some experiments, cells were treated with the indicated drug prior to assaying luciferase activity. In some experiments, the pGL3–5xMCAT(SV)-49 and PRL-TK were co-transfected into cells with other constructs (500 ng/12 well) and then assayed at the indicated times for luciferase activity.

### Western blotting and qPCR

For Western blotting, cells were lysed in Cell Lysis Buffer (Cell Signaling Technology), and protein concentration was determined by the Pierce^TM^ BCA protein assay kit (Thermo Scientific, 23225). Equal protein (15–30 μg) was then subjected to 10% SDS-PAGE, transferred to nitrocellulose membranes, and assayed by Western blotting. The primary antibodies used are listed in Table S5 along with their source, catalogue number, and dilution. Horseradish peroxidase–conjugated secondary antibodies were used at the following concentrations: goat anti-rabbit IgG (Thermo Scientific), 1:5,000; goat anti-mouse IgG (Thermo Scientific), 1:5,000. Western blotting images were captured using the Fujifilm LAS-3000 gel imager, and band intensities were quantified on nonsaturated images using MultiGauge version 3.0 software. Relative band intensities are listed *below* individual immunoblots and were calculated by normalizing the raw band intensities for each lane to a control sample. To determine the ratio of phosphorylated to total YAP, LATS, or MST, relative band intensities for each total and phosphorylated protein were first normalized to the control sample and then to GAPDH for that membrane, and then the ratio of phosphorylated over total protein was calculated. For qPCR, cells were lysed in TRIzol^TM^ reagent (Invitrogen), and RNA was isolated according to the manufacturer's protocol. One μg of total RNA was reverse-transcribed to produce cDNA template using the First-Strand cDNA synthesis kit (Promega). qPCRs were carried out on 2 μl of cDNA, with 12.5 μl of IQ SYBR Green Supermix (Bio-Rad) and 0.4 μmol of each primer. Primer sequences were as follows: human CTGF (forward, 5′-TGCCATTACAACTGTCCCG-3′; reverse, 5′-CAAGTTCCAGTCTAATGAGTTAATGTC-3′); human CYR61 (forward, 5′-GAACTGGTATCTCCACACGAG-3′; reverse, 5′-GGGATTTCTTGGTCTTGCTG-3′); human ANKRD1 (forward, 5′-GGTGAGACTGAACCGCTATAAG-3′; reverse, 5′-GGCTGTCGAATATTGCTTTGG-3′); human GAPDH (forward, 5′-CGTGGAAGGACTCATGACCA-3′; reverse, 5′-GCCATCACGCCACAGTTTC-3′); mouse CTGF (forward, 5′-CTCCACCCGAGTTACCAATG-3′; reverse, 5′-TGGCGATTTTAGGTGTCCG-3′); mouse CYR61 (forward, 5′-ACCAATGACAACCCAGAGTG-3′; reverse, 5′-AAGTAAATCTGACTGGTTCTGGG-3′); mouse β-actin (forward, 5′-TGTATGAAGGCTTTGGTCTCC-3′; reverse, 5′-GTCTCAAGTCAGTGTACAGGC-3′). qPCRs were run using the MyiQ^TM^ real-time PCR detection system according to the manufacturer's instructions (Bio-Rad). PCR conditions were 94 °C for 3 min, followed by 40 cycles of 94 °C for 30 s, 60 °C for 30 s, and 72 °C for 30 s. For analysis, the -fold change relative to the indicated control sample was calculated using the MyiQ^TM^ software, and either GAPDH or β-actin was used as a reference gene.

### Adhesion assays

Cells were transiently transfected with YAP/TAZ-TEAD transcriptional reporter vectors for 24 h and then rinsed and then incubated in serum-free medium for 24 h. Then cells were trypsinized, and trypsin was quenched by adding an equal volume of 10 mg/ml soybean trypsin inhibitor and incubating at 37 °C, 5% CO_2_ for 3 min. Cells were next pelleted, washed two times with 1× PBS, and then resuspended in serum-free medium and left rocking in suspension for 30 min. Afterward, an equal volume of 2% serum-containing medium was added to the cells to bring them to a final concentration of 1% serum, and the cells were plated onto poly-l-lysine, collagen, or fibronectin-coated plates (see below). An aliquot of cells was collected just prior to plating to serve as the 0-h control sample. Cells were allowed to attach and spread for the times indicated in the figure legends. In some experiments, cells were treated with DMSO or 500 nm dasatinib 1 h after plating to inhibit SFK. ECM-coated plates were prepared as follows. 0.01% poly-l-lysine was added for 5 min and then aspirated, and the plates were allowed to air-dry for 2 h. Fibronectin (10 μg/ml) or collagen (30 μg/ml) was added for 1 h and then removed, and plates were rinsed with 1× PBS. After coating, all plates were blocked with heat-inactivated 1% BSA in PBS for 1 h.

### Immunoprecipitation

Cells were seeded onto 10-cm tissue culture plates at 2 × 10^6^ cells in full-growth medium, and 24 h later, they were transiently transfected with 2.5 μg of FLAG-tagged Cool-1 or FLAG-tagged GIT1 using Lipofectamine 3000 (Invitrogen). 24 h later, the cells were treated as indicated in the figure legends and then lysed for 30 min on ice with 1,000 μl of lysis buffer (100 mm NaCl, 300 mm sucrose, 3 mm MgCl_2_, 10 mm PIPES, pH 6.8, 5% IGEPAL CA-630, 1 mm EDTA) containing Halt^TM^ protease and phosphatase inhibitor mixture (Thermo Scientific, Waltham MA). Plates were then scraped to collect lysate and centrifuged (14,000 rpm) at 4 °C for 10 min to remove insoluble material. Equal total protein (0.75–1 mg) was then diluted to a 1-ml total volume with lysis buffer and immunoprecipitated as described below. Prior to IPs, one-tenth of each diluted lysate was set aside for Western blots of whole-cell lysates. The lysates were incubated with the indicated antibodies at 1:200 for 2 h at 4 °C with constant agitation, and then 100 μl of the appropriate equilibrated SureBeads^TM^ magnetic beads (Bio-Rad, 161-4013) were added, and the lysates were incubated at 4 °C overnight with constant agitation. Beads were then collected by magnet and washed twice with lysis buffer and then eluted with 50 μl of 20 mm glycine, pH 2.0. Elution was neutralized with 5 μl of PBS (pH 7.4) and assayed by Western blotting as described above. As indicated in the figure legends, some samples were treated with 1 unit of calf intestinal alkaline phosphatase (New England Biolabs, M0290S) for 30 min prior to Western blotting. Although not shown, optimization immunoprecipitation experiments, which included control IgG and beads-only IPs, were performed for each antibody prior to these experiments.

### In vivo assays

The Albany Medical College Institutional Animal Care and Use Committee approved all mouse studies. Mice were housed in specific pathogen-free conditions in the Albany Medical College Animal Resources Facility, which is licensed by the United States Department of Agriculture and the New York State Department of Health, Division of Laboratories and Research, and is accredited by the Association for Assessment and Accreditation of Laboratory Animal Care International. These studies used either immunocompromised mice (NOD/Scid (NOD/MrkBomTac-*Prkdc^scid^*, Taconic) and NSG (NOD.Cg-*Prkdc^scid^ Il2rg^tm1Wj^l/*SzJ, Jackson Laboratories)) or BALB/c mice (Taconic). To assay tumor formation, tumor size, and overall survival, mice were injected subcutaneously with the indicated number of cells, and then tumor formation was monitored. Tumor volume was assayed at least twice per week by measuring the width and length of each tumor using Vernier calipers. Tumor volume was estimated using the equation, volume = (width × length^2^)/2, and mice were euthanized when the estimated tumor volume reached ∼1,400 cm^3^ (typically 1.5–2 g). After euthanasia, primary tumors were removed and weighed. In some cases, mice were euthanized sooner if they became moribund or the tumors were ulcerated. Survival plots show the percentage of mice that developed tumors and were still alive at each date postinjection. To assay metastasis formation, fluorescently labeled tumor cells were injected into the lateral tail veins of mice, and after 3–5 weeks, mice were euthanized and lung metastases were counted using a fluorescent stereomicroscope (Olympus SZX9). In experiments where metastatic burden was high, images were taken of each lung lobe using the fluorescent stereomicroscope and Lumen*era* Infinity3S camera, and metastases were counted using Cell Profiler and the “ExampleTumor” module (85). For tail vein metastasis assays using 4T1 cells, the cells stably expressed tomato, but metastases were not bright enough to be accurately counted, so lung metastatic burden was measured using qPCR for the stably integrated tomato gene. For this, lungs were extracted, and genomic DNA was isolated using phenol-chloroform extraction. qPCR (described above) was then used to quantify the relative amount of tomato gene and mouse genomic *GAPDH* in the lungs of each mouse. Primer sequences were as follows: tomato (forward, 5′- AAGCTGAAGGTGACCAAGG-3′; reverse, 5′-TTGGAGCCGTACATGAACTG-3′); mouse genomic *GAPDH* (forward, 5′-CCACTCACGGCAAATTCAAC-3′; reverse, 5′-CTCCACGACATACTCAGCAC-3′). All mice are represented as a -fold change from one control shRNA-expressing mouse, and genomic *GAPDH* was used as the reference gene. MA2 and A375 mouse experiments included roughly equal numbers of males and females, and we saw the same trends in both sexes and no indication that sex was a variable. For breast cancer experiments with 4T1 cells, we used only female mice.

### Statistics

The statistical test used to determine significance is indicated in the figure legends. All scatter plots show mean ± S.D. Statistical analysis was done in either Excel (*t* tests) or Prism (all other tests).

## Author contributions

J. M. L., Y. X., E. N., J. S. A. W., and R. O. H. conceptualization; J. M. L., Y. X., E. N., Z.-G. J., G. M. G., J. S. A. W., and S. K. data curation; J. M. L., Y. X., E. N., Z.-G. J., G. M. G., S. K., J. S. A. W., and R. O. H. formal analysis; J. M. L. and R. O. H. supervision; J. M. L. and R. O. H. funding acquisition; J. M. L., Y. X., and E. N. validation; J. M. L., Y. X., E. N., Z.-G. J., G. M. G., S. K., J. S. A. W., and J. S. A. W. investigation; J. M. L. visualization; J. M. L., Y. X., E. N., Z.-G. J., G. M. G., S. K., J. S. A. W., and R. O. H. methodology; J. M. L. writing-original draft; J. M. L. and R. O. H. project administration; J. M. L., Y. X., E. N., J. S. A. W., and R. O. H. writing-review and editing.

## Supplementary Material

Supporting Information

## Footnotes

3 The abbreviations used are:

YAP: Yes-associated protein
TAZ: transcriptional co-activator with PDZ-binding motif
MST1: mammalian sterile 20-like kinase 1
MST2: mammalian sterile 20-like kinase 2
SAV1: Salvador homolog 1
LATS1: large tumor suppressor homolog 1
LATS2: large tumor suppressor homolog 2
MOB1A: MOB kinase activator 1A
MOB1B: MOB kinase activator 1B
TEAD: TEA domain family member
GIT1: GTPase-activating protein GIT ArfGAP 1
PI3K: phosphoinositide 3 kinase
CTGF: connective tissue growth factor
CYR61: cysteine-rich angiogenic inducer 61
SFK: SRC family kinase
ANKD1: ankyrin repeat domain 1
JNK: c-Jun N-terminal kinase
FAK: focal adhesion kinase
qPCR: quantitative PCR
IP: immunoprecipitation
ANOVA: analysis of variance.
