# Supplementary material for: SRC tyrosine kinase activates the YAP/TAZ axis and thereby drives tumor growth and metastasis
Source: J Biol Chem. 2018 Dec 17;294(7):2302–17. doi: 10.1074/jbc.RA118.004364 (PMC6378979; doi:10.1074/jbc.RA118.004364)
Supplement: Supporting Information [file supp_294_7_2302__index.html]

SRC tyrosine kinase activates the YAP/TAZ axis and thereby drives tumor growth and metastasis — SRC drives YAP/TAZ-mediated tumor growth & metastasis — SRC tyrosine kinase activates the YAP/TAZ axis and thereby drives tumor growth and metastasis — SRC drives YAP/TAZ-mediated tumor growth and metastasis — Supporting Information 

# SRC tyrosine kinase activates the YAP/TAZ axis and thereby drives tumor growth and metastasis

## Supporting Information

- Supporting Information (to be published online) - Supporting Information with Figures
- Supplemental Tables 2-5 - Excel file with supplemental tables 2-5
