# Supplementary material for: SRC tyrosine kinase activates the YAP/TAZ axis and thereby drives tumor growth and metastasis
Source: J Biol Chem. 2018 Dec 17;294(7):2302–17. doi: 10.1074/jbc.RA118.004364 (PMC6378979; doi:10.1074/jbc.RA118.004364)
Supplement: Supporting Information [file supp_RA118.004364_138692_2_supp_251341_pjvsgw.pdf]

SRC tyrosine kinase activates the YAP/TAZ axis and thereby drives tumor growth and metastasis

John M. Lamar, Yuxuan, Xiao, Emily Norton, Zhi-Gang Jiang, Genevieve M. Gerhard, Simrin Kooner, and Richard O. Hynes

Supporting Information:

Included Supporting Information**:**

- Supplemental Figures 1-5 (and legends),
- Supplemental Table 1 (and legends)
- Supplemental Tables 2-5 (**attached as separate excel file**)
- Legends Supplemental Tables 2-5
- References for Supporting Information

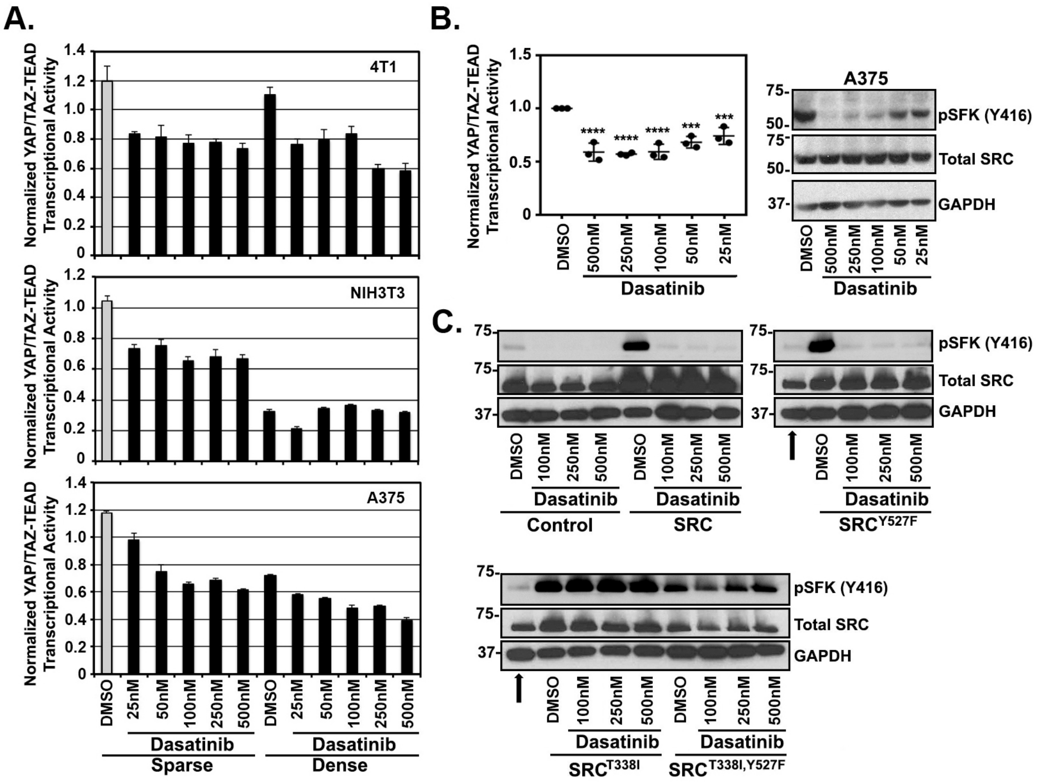

Supplemental Figure 1. Dasatinib reduces YAP/TAZ-TEAD activity in a dose-dependent manner.

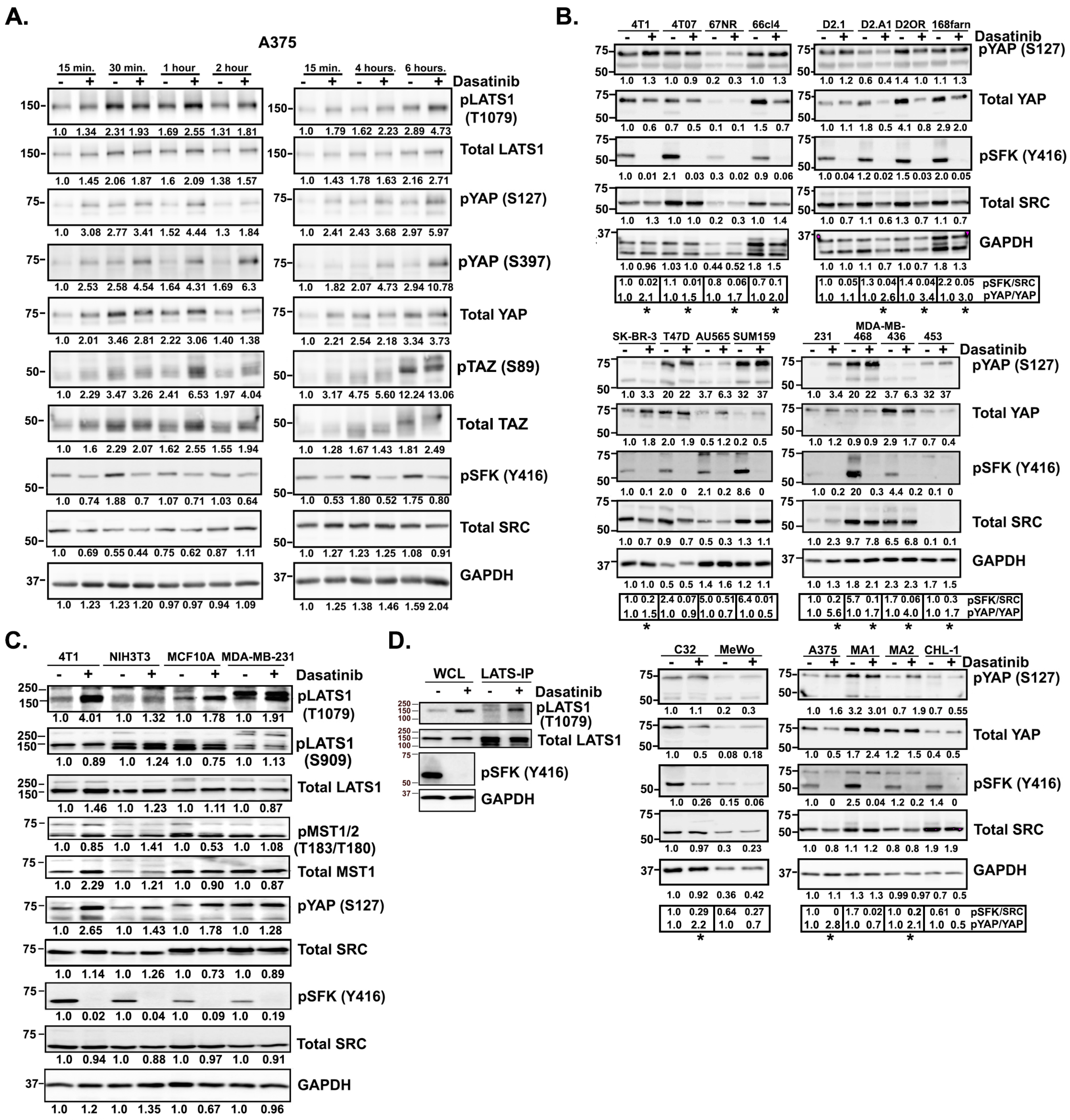

Supplemental Figure 2. SRC inhibition increases YAP, TAZ, and LATS phosphorylation.

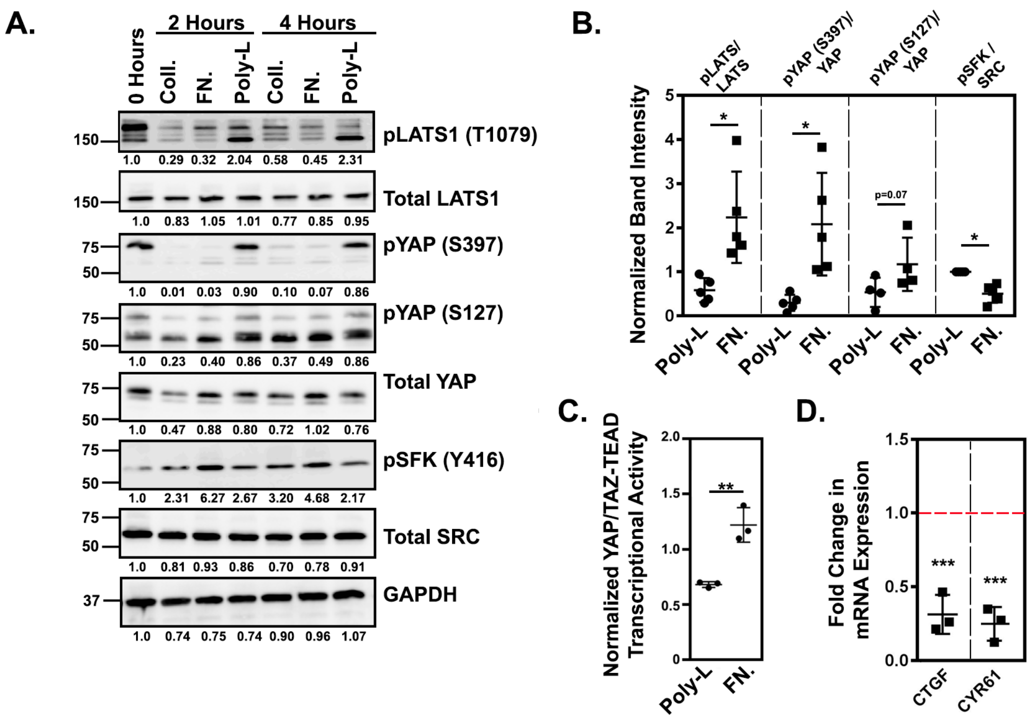

Supplemental Figure 3. Cell-ECM adhesion activates SRC and promotes YAP/TAZ activity.

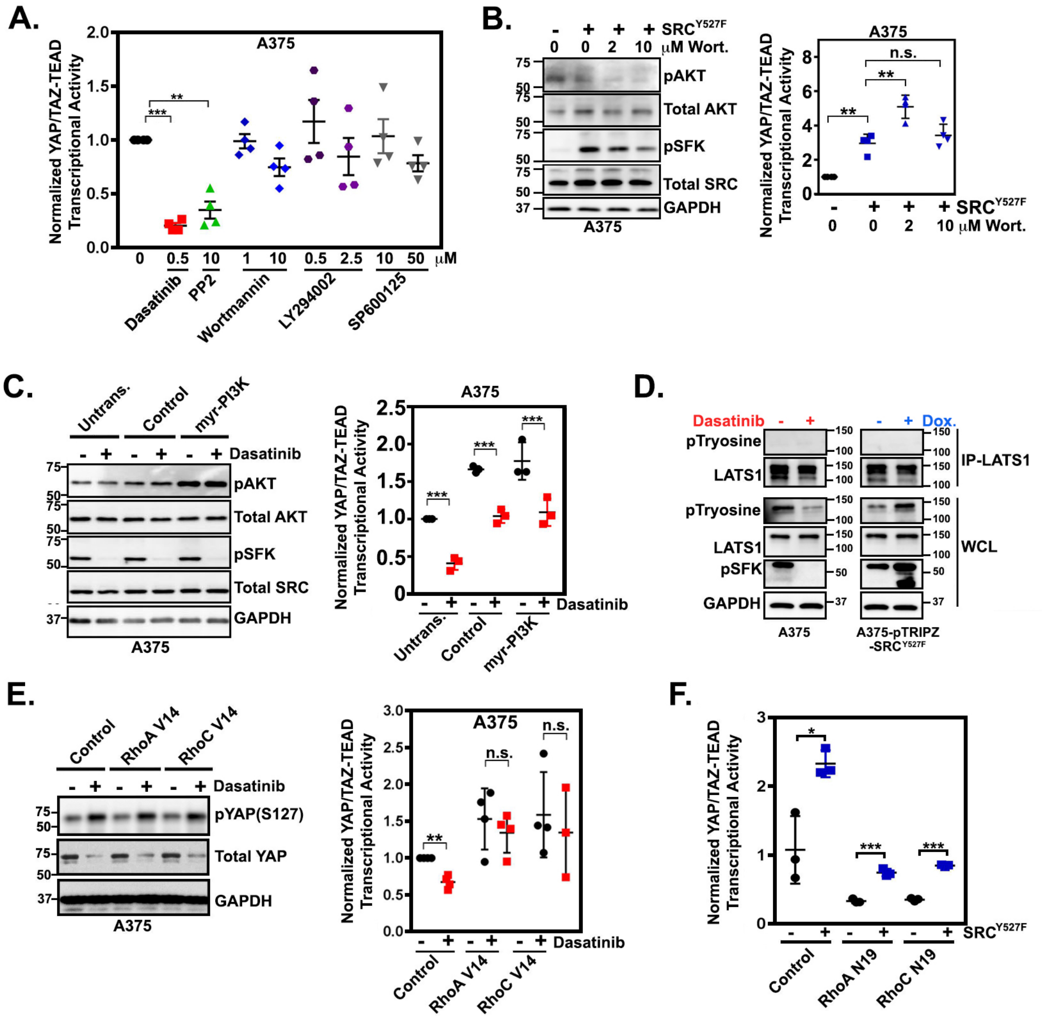

Supplemental Figure 4. Alteration of PI3 Kinase or Rho signaling does not significantly influence SRC-mediated YAP/TAZ activation.

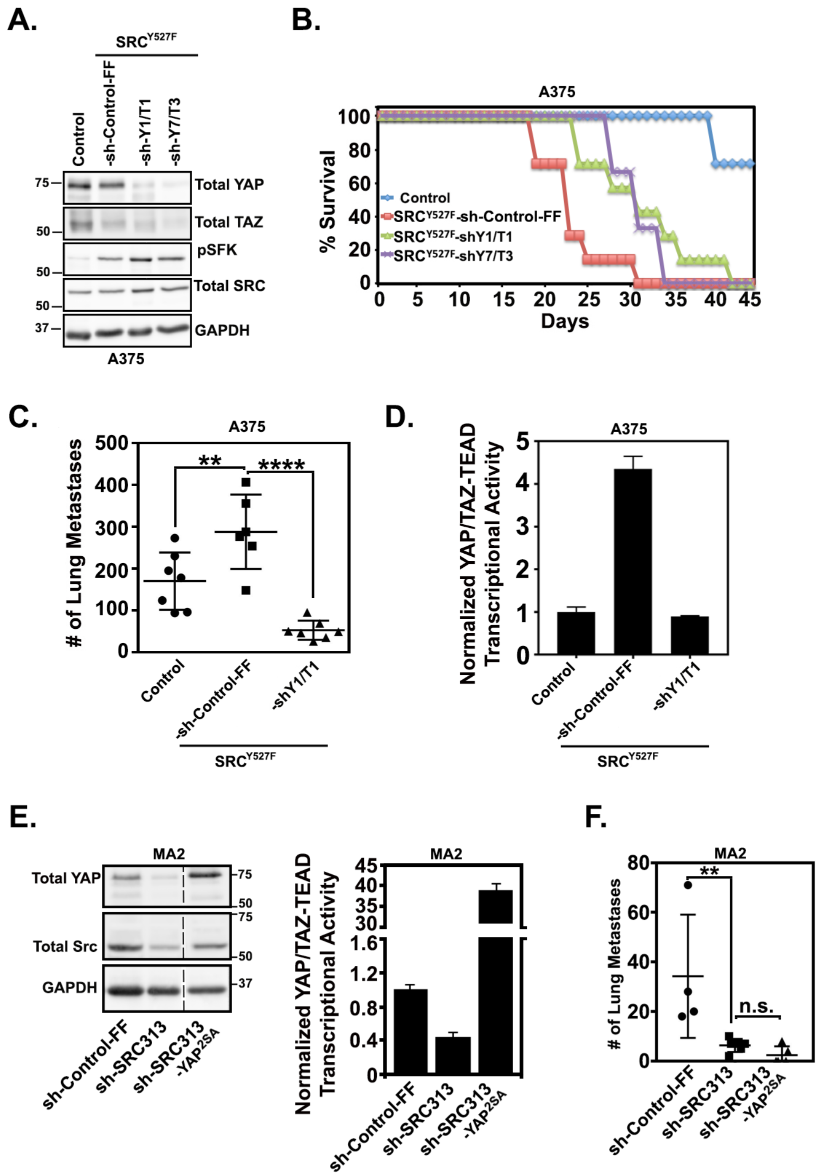

Supplemental Figure 5. SRC-mediated YAP/TAZ activation promotes tumor growth and metastasis.

Injected Cells (Mouse #)	Tumor Volume (cm3) on Day 20	Tumor Volume (cm3) on Day 26	Tumor Volume (cm3) on Day 31	Tumor Volume (cm3) Day 35	Tumor Volume (cm3) on Day 40	Tumor Volume (cm3) on Day 45	Tumor Volume (cm3) on Day 49
A375-Control (1)	4	32	108	405	700	800	2816
A375-Control (2)	4	32	320	252	640	1440	
A375-Control (3)	0	32	364.5	446	1152	2176	
A375-Control (4)	0	4	4	288	320	700	850
A375-SRC-Y527F (1)	2432						
A375-SRC-Y527F (2)	2601						
A375-SRC-Y527F (3)	1152	1774.5					
A375-SRC-Y527F (4)	294	700	1440				
A375-SRC-Y527F (5)	4	32	32	87.5	365	108	455
A375-SRC-Y527F-Y1/T1 (1)	1912.5						
A375-SRC-Y527F-Y1/T1 (2)	4	269.5	384	750	1568		
A375-SRC-Y527F-Y1/T1 (3)	4	108	126	180	600	847	2560
A375-SRC-Y527F-Y1/T1 (4)	0	4	4	126	405	600	1568
A375-SRC-Y527F-Y1/T1 (5)	0	0	0	0	0	0	0

Supplemental Table 1: SRC promotes tumor growth and reduces survival in a YAP/TAZ-dependent manner.

Supplemental Figure 1. Dasatinib reduces YAP/TAZ-TEAD activity in a dose-dependent manner.
(A) The indicated cell lines plated at sparse or high density were treated with control DMSO or the indicated doses of Dasatinib for 6.5 hours and then assayed for YAP/TAZ-TEAD transcriptional activity (n=4 replicates from 1 experiment).
(B) A375 cells were treated with control DMSO or the indicated doses of Dasatinib for 6.5 hours then assayed by Western blot **(right panel)** or for YAP/TAZ-TEAD transcriptional activity **(left panel)**. The scatter plot shows mean $\pm$ S.D. where each dot is an independent experiment. Statistical significance was tested using one-way ANOVA with Dunnett's multiple comparisons test.
(C) Western blots for one representative experiment that yielded the data in **Figure 2A**. The arrows indicate the control DMSO sample from the first blot that was also loaded on the other two blots to allow comparisons between blots.

Supplemental Figure 2. SRC inhibition increases YAP, TAZ, and LATS phosphorylation.
(A-C) The indicated cells were treated with control DMSO or 500nM Dasatinib for the indicated times and then assayed by Western blot. Relative band intensities are indicated under each blot. In **B** the ratios of pYAP/YAP and pSRC/SRC are also indicated. * indicates lines that showed increased pYAP/YAP levels following Dasatinib treatment.
(D) Western blots were performed on whole cell lysate (WCL) or following immunoprecipitation (IP) with a LATS antibody for total and phosphorylated LATS, pSFK, and GAPDH.

Supplemental Figure 3. Cell-ECM adhesion activates SRC and promotes YAP/TAZ activity.
(A) Representative set of Western blots from A375 cells following adhesion assays (see Methods). Band intensities, normalized to the sample collected at 0 hours, are indicated under each blot.
(B) Quantification of 3 separate experiments performed as in **A**. For each, band intensities for the 4-hour fibronectin and poly-l-lysine samples were normalized to the 0 hour sample, and then to GAPDH, and the ratio of phosphorylated to total protein was calculated and plotted.
(C, D) Adhesion assays with A375 cells plated on fibronectin or poly-l-lysine for 6.5 hours and assayed for YAP/TAZ-TEAD transcriptional activity **(C)** or by qPCR **(D)**. Samples in **C&D** were normalized to a 0 hour sample.
Statistical Analyses: Scatter plots show mean $\pm$ S.D. where each dot is an independent experiment. Statistical significance was tested using Student's T-test. * $p \leq 0.05$, ** $p \leq 0.01$, *** $p \leq 0.001$.

Supplemental Figure 4. Alteration of PI3 Kinase or Rho signaling does not significantly influence SRC-mediated YAP/TAZ activation.
(A) A375 cells treated for 6.5 hours with the indicated doses of DMSO (0), Dasatinib, PI3Kinase inhibitors (Wortmannin or LY294002) or a JNK inhibitor (SP600125), and then assayed for YAP/TAZ-TEAD transcriptional activity.
(B) A375 cells co-transfected overnight with the YAP/TAZ-TEAD reporter constructs and control empty vector (-) or SRC^{Y527F} (+) were treated with DMSO or Wortmannin and then assayed by Western blot 1 hour later **(left)** or for YAP/TAZ-TEAD transcriptional activity 6.5 hours later **(right)**.
(C) A375 cells co-transfected overnight with the YAP/TAZ-TEAD reporter constructs and nothing, (Untrans.), empty vector (Control), or myr-PI3 Kinase were treated with DMSO (-) or 500nM Dasatinib for 6.5 hours and then assayed by Western blot **(left)** or for YAP/TAZ-TEAD transcriptional activity **(right)**.
(D) A375 cells treated with DMSO (-) or 500nM Dasatinib for 1 hour **(left)**, or A375-pTRIPZ- SRC^{Y527F} cells treated with water (-) or 0.5μg/ml doxycycline (+) for 12 hours **(right)** were lysed and Western blots were performed on whole cell lysate (WCL) or after immunoprecipitation with an anti-LATS1 antibody (IP-LATS1). Results are representative of 3 experiments.
(E) A375 cells co-transfected with the YAP/TAZ-TEAD reporter constructs and control empty vector or activated Rho constructs (RhoA-V14 and RhoC-V14) were treated with DMSO (-) or 500nM Dasatinib

for 6.5 hours and then assayed by Western blot **(left)** or for YAP/TAZ-TEAD transcriptional activity **(right)**.

(F) A375 cells stably expressing control empty vector or SRC^{Y527F} were co-transfected with the YAP/TAZ-TEAD reporter constructs and control empty vector or dominant-negative RhoA-N19 or RhoC-N19 and 24 hours later were assayed for YAP/TAZ-TEAD transcriptional activity.

Statistical Analyses: Scatter plots show mean $\pm$ S.D. where each dot is an independent experiment (in **A** n=4 wells from 2 independent experiments). Statistical significance was tested using one-way ANOVA with Dunnett's multiple comparisons **(A)**, Student's T-test with Bonferroni correction for multiple comparisons **(B, E, & F)**, and one-way ANOVA with Tukey's multiple comparisons test **(C)**. n.s.= p> 0.05, * p$\leq$ 0.05, ** p$\leq$ 0.01, and *** p$\leq$ 0.001

Supplemental Figure 5. SRC-mediated YAP/TAZ activation promotes tumor growth and metastasis.

(A) Western blot on A375 cells expressing control empty vector, SRC^{Y527F}, or SRC^{Y527F} and either a control shRNA (sh-Control-FF) or tandem YAP and TAZ shRNAs (shY1/T1 or shY7/T3).

(B&C) $5x10^5$ cells from **A** were injected either subcutaneously **(B)**, or into the lateral the tail veins **(C)** of NOD/Scid mice and then mouse survival was assayed **(B)** and lung metastases were counted after 5 weeks **(C)**.

(D) Cells from **A** were assayed for YAP/TAZ-TEAD transcriptional activity (n=4 replicates from 1 experiment done at the time of injections in **B&C**).

(E) MA2 cells stably expressing a control shRNA (sh-Control-FF), a SRC shRNA (sh-SRC313), or SRC shRNA and YAP^{2SA} were assayed by Western blot **(left)** or for YAP/TAZ-TEAD transcriptional activity **(right)** (n=4 replicates from 1 experiment done at the time of injection).

(F) Lung metastases were counted 21 days after injection of cells from **E** into the lateral tail vein of NOD/Scid mice.

Statistical Analyses: Scatter plots show mean $\pm$ S.D. where each dot is a mouse. Statistical significance was tested using one-way ANOVA with Dunnett's multiple comparisons test. n.s.= p> 0.05, ** p$\leq$0.01, and **** p$\leq$ 0.0001.

Supplemental Table 1: SRC promotes tumor growth and reduces survival in a YAP/TAZ-dependent manner. Shown are the tumor volumes estimated as described in the Methods for each mouse injected in **Figure 7C**. The number of days post injection is listed for each measurement (column). Mice were euthanized when the tumor volume reached ~1400 cm^3. If a mouse was euthanized, all subsequent measurements will be gray boxes.

Supplemental Table 2: Cell lines and Sources. Listed are all cell lines used in these studies along with their species, cell type, source, and culture media. Citations refer to: **[1]**(1), **[2]** (2), **[3]**(3).

Supplemental Table 3: List of Vectors and their Source. The top table shows existing, purchased, or gifted vectors used in this study and their source. The bottom table shows all new vectors that were cloned for this study and lists the source vector for the insert and the backbone. Citations refer to: **[1]** (4) **[2]** (5) **[3]** (6) **[4]** (7) **[5]** (8) **[6]** (9) **[7]** (10) **[8]** (11) **[9]** (12) **[10]** (13) **[11]** (14) **[12]** (15) **[13]** (16) **[14]** (17) **[15]** (18) **[16]** (19) **[17]** (20) **[18]** (21).

Supplemental Table 4: List of miR30-based shRNAs. Listed are the sequences of the 97-mers used to clone the miR30-based shRNAs targeting human SRC, YAP, TAZ, and the control shRNA.

Supplemental Table 5: Antibodies used. The table lists each antibody used along with their species, source, catalogue number, and dilution.

1. Labelle, M., Begum, S., and Hynes, R. O. (2011) Direct signaling between platelets and cancer cells induces an epithelial-mesenchymal-like transition and promotes metastasis. *Cancer cell* **20**, 576-590
2. Aslakson, C. J., and Miller, F. R. (1992) Selective events in the metastatic process defined by analysis of the sequential dissemination of subpopulations of a mouse mammary tumor. *Cancer Res* **52**, 1399-1405
3. Xu, L., Begum, S., Hearn, J. D., and Hynes, R. O. (2006) GPR56, an atypical G protein-coupled receptor, binds tissue transglutaminase, TG2, and inhibits melanoma tumor growth and metastasis. *Proc Natl Acad Sci U S A* **103**, 9023-9028
4. Naviaux, R. K., Costanzi, E., Haas, M., and Verma, I. M. (1996) The pCL vector system: rapid production of helper-free, high-titer, recombinant retroviruses. *J Virol* **70**, 5701-5705
5. Lamar, J. M., Stern, P., Liu, H., Schindler, J. W., Jiang, Z. G., and Hynes, R. O. (2012) The Hippo pathway target, YAP, promotes metastasis through its TEAD-interaction domain. *Proc Natl Acad Sci U S A* **109**, E2441-2450
6. Sena-Esteves, M., Tebbets, J. C., Steffens, S., Crombleholme, T., and Flake, A. W. (2004) Optimized large-scale production of high titer lentivirus vector pseudotypes. *J Virol Methods* **122**, 131-139
7. Reya, T., Duncan, A. W., Ailles, L., Domen, J., Scherer, D. C., Willert, K., Hintz, L., Nusse, R., and Weissman, I. L. (2003) A role for Wnt signalling in self-renewal of haematopoietic stem cells. *Nature* **423**, 409-414
8. Mahoney, W. M., Jr., Hong, J. H., Yaffe, M. B., and Farrance, I. K. (2005) The transcriptional co-activator TAZ interacts differentially with transcriptional enhancer factor-1 (TEF-1) family members. *Biochem J* **388**, 217-225
9. Khosravi-Far, R., White, M. A., Westwick, J. K., Solski, P. A., Chrzanowska-Wodnicka, M., Van Aelst, L., Wigler, M. H., and Der, C. J. (1996) Oncogenic Ras activation of Raf/mitogen-activated protein kinase-independent pathways is sufficient to cause tumorigenic transformation. *Mol Cell Biol* **16**, 3923-3933
10. Hao, H., Muniz-Medina, V. M., Mehta, H., Thomas, N. E., Khazak, V., Der, C. J., and Shields, J. M. (2007) Context-dependent roles of mutant B-Raf signaling in melanoma and colorectal carcinoma cell growth. *Mol Cancer Ther* **6**, 2220-2229
11. Zhao, J. J., Liu, Z., Wang, L., Shin, E., Loda, M. F., and Roberts, T. M. (2005) The oncogenic properties of mutant p110alpha and p110beta phosphatidylinositol 3-kinases in human mammary epithelial cells. *Proc Natl Acad Sci U S A* **102**, 18443-18448
12. Espanel, X., and Sudol, M. (2001) Yes-associated protein and p53-binding protein-2 interact through their WW and SH3 domains. *J Biol Chem* **276**, 14514-14523
13. Zhao, B., Li, L., Lu, Q., Wang, L. H., Liu, C. Y., Lei, Q., and Guan, K. L. (2011) Angiomotin is a novel Hippo pathway component that inhibits YAP oncoprotein. *Genes Dev* **25**, 51-63
14. Johnson, K. C., Kissil, J. L., Fry, J. L., and Jacks, T. (2002) Cellular transformation by a FERM domain mutant of the Nf2 tumor suppressor gene. *Oncogene* **21**, 5990-5997
15. Zhang, X. H., Wang, Q., Gerald, W., Hudis, C. A., Norton, L., Smid, M., Foekens, J. A., and Massague, J. (2009) Latent bone metastasis in breast cancer tied to Src-dependent survival signals. *Cancer cell* **16**, 67-78

16. Lei, Q. Y., Zhang, H., Zhao, B., Zha, Z. Y., Bai, F., Pei, X. H., Zhao, S., Xiong, Y., and Guan, K. L. (2008) TAZ promotes cell proliferation and epithelial-mesenchymal transition and is inhibited by the hippo pathway. *Mol Cell Biol* **28**, 2426-2436
17. Zhao, B., Wei, X., Li, W., Udan, R. S., Yang, Q., Kim, J., Xie, J., Ikenoue, T., Yu, J., Li, L., Zheng, P., Ye, K., Chinnaiyan, A., Halder, G., Lai, Z. C., and Guan, K. L. (2007) Inactivation of YAP oncoprotein by the Hippo pathway is involved in cell contact inhibition and tissue growth control. *Genes Dev* **21**, 2747-2761
18. Oka, T., Mazack, V., and Sudol, M. (2008) Mst2 and Lats kinases regulate apoptotic function of Yes kinase-associated protein (YAP). *J Biol Chem* **283**, 27534-27546
19. Lin, Y., Khokhlatchev, A., Figeys, D., and Avruch, J. (2002) Death-associated protein 4 binds MST1 and augments MST1-induced apoptosis. *J Biol Chem* **277**, 47991-48001
20. Mayhew, M. W., Webb, D. J., Kovalenko, M., Whitmore, L., Fox, J. W., and Horwitz, A. F. (2006) Identification of protein networks associated with the PAK1-betaPIX-GIT1-paxillin signaling complex by mass spectrometry. *J Proteome Res* **5**, 2417-2423
21. Manabe, R., Kovalenko, M., Webb, D. J., and Horwitz, A. R. (2002) GIT1 functions in a motile, multi-molecular signaling complex that regulates protrusive activity and cell migration. *J Cell Sci* **115**, 1497-1510
